# Radical change of apoptotic strategy following irradiation during later period of embryogenesis in medaka (*Oryzias latipes*)

**DOI:** 10.1371/journal.pone.0201790

**Published:** 2018-08-03

**Authors:** Takako Yasuda, Yuta Ishikawa, Noriko Shioya, Kazusa Itoh, Miyuki Kamahori, Kento Nagata, Yoshiro Takano, Hiroshi Mitani, Shoji Oda

**Affiliations:** 1 Department of Integrated Biosciences, Graduate School of Frontier Sciences, the University of Tokyo, Kashiwa, Chiba, Japan; 2 Section of Biostructural Science, Graduate School of Medical and Dental Sciences, Tokyo Medical and Dental University, Tokyo, Japan; Northwestern University Feinberg School of Medicine, UNITED STATES

## Abstract

Induction of apoptosis in response to various genotoxic stresses could block transmission of teratogenic mutations to progeny cells. The severity of biological effects following irradiation depends on the stage at which embryos are irradiated during embryogenesis. We reported previously that irradiation of medaka embryos 3 days post fertilization (dpf) with 10 Gy of gamma rays induced high incidence of apoptotic cells in the mid-brain, however, the embryos hatched normally and developed without apparent malformations. To determine the severity of biological effects following irradiation during a later period of embryogenesis, embryos of various developmental stages were irradiated with 15 Gy of gamma rays and examined for apoptotic induction at 24 h after irradiation in the brain, eyes and pharyngeal epithelium tissues, which are actively proliferating and sensitive to irradiation. Embryos irradiated at 3 dpf exhibited many apoptotic cells in these tissues, and all of them died due to severe malformations. In contrast, embryos irradiated at 5 dpf showed no apoptotic cells and subsequently hatched without apparent malformations. Embryos irradiated at 4 dpf had relatively low numbers of apoptotic cells compared to those irradiated at 3 dpf, thereafter most of them died within 1 week of hatching. In adult medaka, apoptotic cells were not found in these tissues following irradiation, suggesting that apoptosis occurs during a restricted time period of medaka embryogenesis throughout the life. No apoptotic cells were found in irradiated intestinal tissue, which is known to be susceptible to radiation damage in mammals, whereas many apoptotic cells were found in proliferating spermatogonial cells in the mature testis following irradiation. Taken together, with the exception of testicular tissue, the results suggest a limited period during medaka embryogenesis in which irradiation-induced apoptosis can occur.

## Introduction

The mammalian embryo and fetus have been shown to be highly radiosensitive during the entire period of prenatal development. The developing central nervous system (CNS) is substantially more susceptible to induced teratogenic malformations than other embryonic and fetal tissues due to structural complexity, long developmental period and the vulnerability of the undifferentiated neural cells compared to developed neurons [1–4]. The formation of most human organs is largely completed by 8 weeks after ovulation and then, a rapid increase in the number of neurons occurs and the development of the cerebral cortex begins during the period of 8–15 weeks after ovulation. Epidemiological studies of prenatally exposed atomic-bomb survivors in Hiroshima and Nagasaki demonstrated the harmful effects of radiation on the developing human brain during the 8–15 weeks after ovulation, including the occurrence of severe mental retardation, reduced intelligence scores and poorer school performance [5–8]. These detrimental effects on the irradiated developing CNS were associated with cell death, changes in cellular differentiation and neural migration. Recent clinical studies of CNS pathologies in children demonstrated that exposure to radiation therapy resulted in a higher incidence of secondary brain tumors due to a greater susceptibility to irradiation compared to adult patients who had undergone radiation therapy [9–11].

The medaka has been shown to be an excellent model for studies on the developing CNS of vertebrates, since their transparency and the small size of embryos enable examination of morphological abnormalities with greater efficiency than mammalian embryos [12, 13]. In addition, morphogenesis in medaka embryonic brain is slower than zebrafish (*Danio rerio*), another popular laboratory fish model [14]. It has been suggested that studies on the medaka will provide more detailed information about the effects of ionizing radiation on the developing vertebrate embryonic brain. Moreover, previous studies have revealed the spatiotemporal changes in irradiation-induced apoptosis and phagocytosis of apoptotic fragments by microglia in the developing medaka CNS following irradiation [15, 16]. We have shown that exposure of medaka to 10 Gy of gamma-ray irradiation at 3 days post fertilization (dpf) induced many apoptotic cells, mainly in the marginal area of the optic tectum (OT). Medaka embryos at 3 dpf corresponds to developmental stage 28, according to Iwamatsu staging [17], when neural cells proliferate actively in the OT [18]. Irradiated embryos hatched normally and developed without apparent malformations although histopathological examinations showed they had smaller-sized brains and eyes than control embryos [19–21]. Previous studies on the effects of irradiation on medaka embryos demonstrated that viability changed drastically depending on the embryonic stage at which they were irradiated. Significant severe effects were not induced when embryos were irradiated beyond developmental stage 30 (4 dpf) [22, 23].

In this study, to determine the severity of biological effects following irradiation throughout the lifespan of medaka, we investigated apoptotic induction in actively proliferating tissues at various developmental stages during later period of embryogenesis and adults at 24 h after irradiation. The results indicated that irradiation-induced apoptosis occurred during a limited period of embryogenesis, corresponding to 3 dpf and 4 dpf, with the exception of testicular tissue of adult medaka, in which apoptosis occurred throughout the lifespan.

## Materials and methods

### Ethics

This research was conducted using protocols approved by the Animal Care and Use Committee of the University of Tokyo (permit number: C-09-01). All surgeries on embryos and adult medaka were performed using chilling or 0.02% (w/v) MS222 solution as anesthesia, and all efforts were made to minimize suffering.

### Fish and embryos

An Hd-rR inbred strain of medaka (*Oryzias latipes*), established from a southern population [24], was used in this study. The fish were kept at 26–28°C under a 14 h light and 10 h dark cycle, and fed on a powdered diet (TetraMin, Tetra Werke, Melle, Germany) and brine shrimp (*Artemia franciscana*) three times per day. Female medaka spawn eggs every morning. Egg clusters were collected and rubbed between two small pieces of paper towel to remove filaments on the chorion; the isolated eggs were then incubated in a petri dish filled with 7 ml of distilled water containing 10^−5^% (w/v) methylene blue at 26–28°C. The developmental stages of embryos were described by Iwamatsu [17].

### Irradiation

Embryos and adult medaka were irradiated with gamma rays emitted by ^137^Cs (Gammacell 3000Elan, MDS Nordion, Ottawa, Canada) at a dose rate of 7.3 Gy/min at room temperature (RT) in a plastic tub containing water.

### Detection of apoptotic cells

Acridine orange (AO) (Sigma-Aldrich, St. Louis, MO, USA), a single-strand DNA intercalating vital dye, selectively stains the nuclei of apoptotic cells without labeling necrotic cells [25, 26]. To confirm the frequency of irradiation-induced apoptotic cells during later embryogenesis, we prepared more than five irradiated samples and sham controls in each developmental stage, including 3 dpf, 4 dpf and 5 dpf. The irradiated embryos were stained with AO (17 μg/ml) as previously described [20, 27]. The AO stained cells were detected in the embryos using a fluorescence microscope (BX50, Olympus, Tokyo, Japan) with an appropriate filter (U-MGFPHQ, Olympus, Tokyo, Japan), and 460–480 nm excitation and 495–540 nm emission wavelengths. Images were captured with a digital camera (DFC7000T, Leica, Wetzlar, Germany).

### Histological analyses with light microscopy and electron microscopy

Embryos and adult medaka were anesthetized by chilling or 0.02% (w/v) MS222 solution. For histological analyses in each experimental group, we prepared three irradiated samples and sham controls. Embryos and dissected tissues of adult medaka were fixed in 4% (w/v) paraformaldehyde in 0.1 M phosphate buffer overnight at 0–4°C. The fixed samples were dehydrated in a graded ethanol series, 100%, 75%, 50% and 25%. Embryos were embedded in plastic resin (Technovit 8100, Heraeus Kulzer, Wehrheim, Germany) and dissected tissues of adult medaka were embedded in paraffin (Parabett, Lot No. 160101 Muto Pure Chemicals Co., Ltd., Tokyo, Japan). Samples embedded in resin or paraffin were sectioned frontally (8 μm thick or 5 μm thick, respectively), as previously described [15, 19, 28]. The embryonic sections were Nissl-stained using cresyl violet for light microscopy. For transmission electron microscopic observations, the embryonic brains were fixed and embedded in plastic resin as previously described [20, 29]. Ultrathin sections were cut, stained with uranyl acetate and lead citrate and examined with a Hitachi H-7500 electron microscope operated at 80 kV (Hitachi Ltd., Tokyo, Japan). For scanning electron microscopic (SEM) observations, dissected small intestine was fixed in Davidson’s fixative solution (22% of a 37% solution of formaldehyde, 33% ethanol, 11.5% glacial acetic acid, and 33.5% distilled H_2_O) overnight at RT, cut lengthwise and examined with a Keyence VE9800 SEM operated at 5 kV (Keyence, Osaka, Japan).

The sections for immunohistochemistry were prepared as previously reported [15]. Briefly, embryonic serial sections (20 μm thick) cut on a cryostat and serial sections of adult tissues (5 μm thick) were blocked by incubation in phosphate-buffered saline (PBS) containing normal goat serum for 30 min at RT, washed in PBS and incubated with a polyclonal anti-cleaved caspase-3 antibody (9661S, Cell Signaling Technology, Danvers, MA, USA) (1:200), with a monoclonal anti-PCNA (PC10) antibody (1:500) (NB500-106, Novus Biologicals, Littleton, CO, USA), and with a polyclonal anti-phospho-histone H3 (Ser10) (anti-P-H3) antibody (06–570, Merck, Darmstadt, Germany) (1:200) for 3 h at RT. The sections for fluorescent images were further incubated with secondary antibody conjugated with Alexa-488 (A11001, Invitrogen, Carlsbad, CA, USA) and counterstained with DAPI. Other sections were incubated with biotin-labeled secondary antibodies and with avidin-biotin-horseradish peroxidase complex (ABC Elite kit, Vector Laboratories, Burlingame, CA, USA) (1:100), developed in 0.05% diaminobenzidine and counterstained with cresyl violet for 20 sec. or with hematoxylin for 5 min. Images were captured with a microscope (BX50, Olympus, Tokyo, Japan) equipped with a digital camera (DFC7000T, Leica, Wetzlar, Germany). The sagittal section of whole-body adult medaka as a high-resolution seamless tiling image was cited from a web browser (https://ds.cc.yamaguchi-u.ac.jp/~vs_08_2p/newpage4.html; image from HdrR_M_221.) [28].

### Detection of pharyngeal epithelium by alizarin red staining

Embryos at the hatching period were anesthetized by chilling and fixed in 4% (w/v) paraformaldehyde in 0.1 M phosphate buffer for 48 h at 4°C, rinsed in a graded ethanol series (100%, 75%, 50% and 25%), followed by 0.5% KOH solution in water. They were stained with 10^−3^% alizarin red/0.5% KOH overnight. They were fixed in 4% paraformaldehyde in 0.1 M phosphate buffer for 30 min at RT and transferred through a 0.5% KOH/glycerol and stored in 100% glycerol [30, 31].

### Quantitative evaluation of anti-P-H3-labeled cells

To evaluate the effects of cell proliferation following 15 Gy gamma-ray irradiation in the developing OT, the number of anti-P-H3-labeled cells/OT were counted in transverse sections at the mid-mesencephalon level. The anti-P-H3-labeled cells were counted under a microscope (BX50, Olympus, Tokyo, Japan). Differences in the numbers of anti-P-H3-labeled cells in the OT between three or four irradiated embryos and sham-controls were analyzed using the student t-test. A p* value < 0.05 was considered statistically significant and p** < 0.01 was considered highly statistically significant.

### Reconstruction and computer visualization of 3D images of medaka intestinal tissue

Reconstruction of 3D images was previously described [16]. Briefly, serial histological sections of PCNA-positive cells were analyzed with a microscope (BX50, Olympus, Tokyo, Japan) and digital camera (DP70, Olympus, Tokyo, Japan). To align the sections, each image was rotated and shifted using Photoshop (Adobe Systems, Mountain View, CA, USA) and the StackReg plug-in for ImageJ (http://imagej.nih.gov/ij/). Following gray-scale conversion, the PCNA-positive cells were segmented manually as colored spots with Photoshop using a pen tablet. Serial gray-scale images and segmented images were stacked as X–Y–Z images on Image J and exported as a Visualization Tool Kit (VTK) file using a KbiVtk plug-in (http://hasezawa.ib.k.u-tokyo.ac.jp/zp/Kbi/ImageJKbiPlugins). Once the VTK files had been exported, reconstructed 3D images of PCNA-positive cells in mature medaka intestine were displayed using a volume rendering method using visualization software ParaView, version 3.10.1 (Sandia National Lab., USA).

## Results

### Radiosensitivity drastically changed in the brain and pharyngeal epithelium during later period of embryogenesis

**Fig 1:**
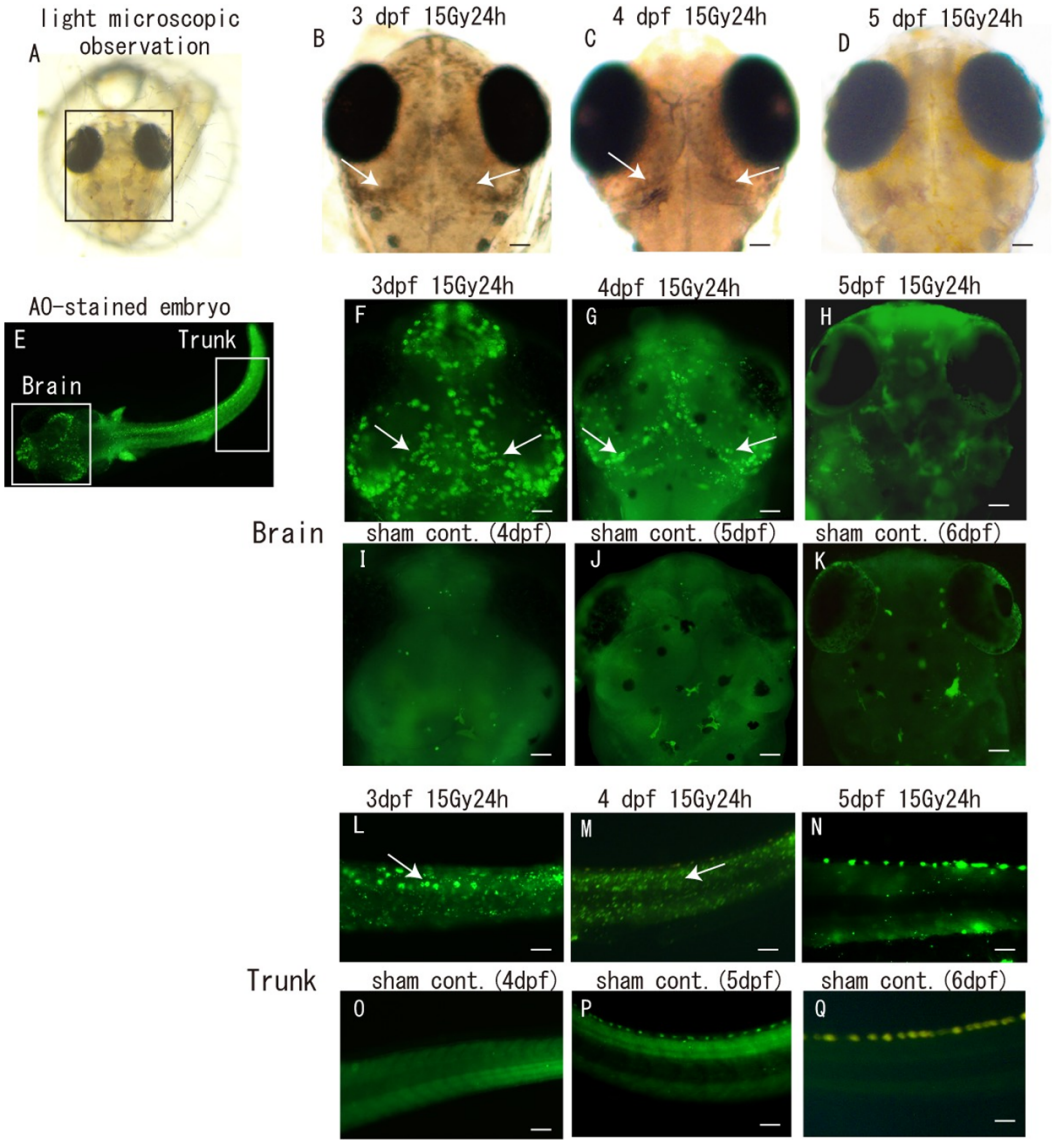
The number of radiation-induced apoptotic cells drastically decreased as embryogenesis proceeded. Embryos at 3, 4 and 5 dpf were irradiated with 15 Gy of gamma rays, and irradiation-induced apoptotic cells in the brain (square areas in A and E) were examined by light microscopy (B–D) and AO assay (F–K) at 24 h after irradiation. Irradiation-induced apoptotic cells in the trunk (rectangular area in E) were examined by AO-assay (L–Q). Opaque dead cells were identified in the marginal area of the OT in irradiated 3 dpf (arrows in B) and 4 dpf (arrows in C) embryonic brains. In irradiated 5 dpf embryonic brain, no opaque dead cells were evident (D). The distribution of AO-stained apoptotic cells in the brain of irradiated 3–5 dpf embryos (arrows in F, G) and in the trunk of irradiated 3–5 dpf embryos (arrows in L, M) were similar manner to those by microscopic observations (arrows in B, C), whereas no apoptotic cells were found in the brain and trunk of sham controls (I-K and O-Q), respectively. Scale bars = 50 μm.

Medaka embryos at 3, 4 and 5 dpf were irradiated with 15 Gy of gamma rays and irradiation-induced apoptotic cells were examined using light microscopy with the acridine orange (AO)-assay 24 h after irradiation. Medaka embryos at developmental stage 28 correspond to early human embryos at approximately 8–15 weeks post-ovulation [18]. By light microscopic observations, many irradiation-induced opaque dead cells were identified in the marginal area of the OT in the irradiated 3 dpf embryonic brain (Fig 1B) and decreased in the irradiated 4 dpf embryonic brain (Fig 1C). No opaque dead cells were found in the irradiated 5 dpf embryonic brain (Fig 1D). Using the AO-assay, the distribution of irradiation-induced apoptotic cells in the brains (Fig 1F–1H) was almost identical to observations using light microscopy in irradiated 3 dpf (Fig 1B), 4 dpf (Fig 1C) and 5 dpf (Fig 1D) embryos respectively, whereas no apoptotic cells were observed in the brain of sham control embryos (Fig 1I–1K). In irradiated part of the body excluding the brain tissue, termed as trunk, the distribution of irradiation-induced apoptotic cells by AO-assay was similar manner as those in the irradiated brain; a lot of apoptotic cells in irradiated 3 dpf embryos (Fig 1L) decreased in irradiated 4 dpf embryos (Fig 1M), and were not present in the irradiated 5 dpf embryos (Fig 1N). In all sham controls of trunk, no apoptotic cells were found, respectively (Fig 1O–1Q).

**Fig 2:**
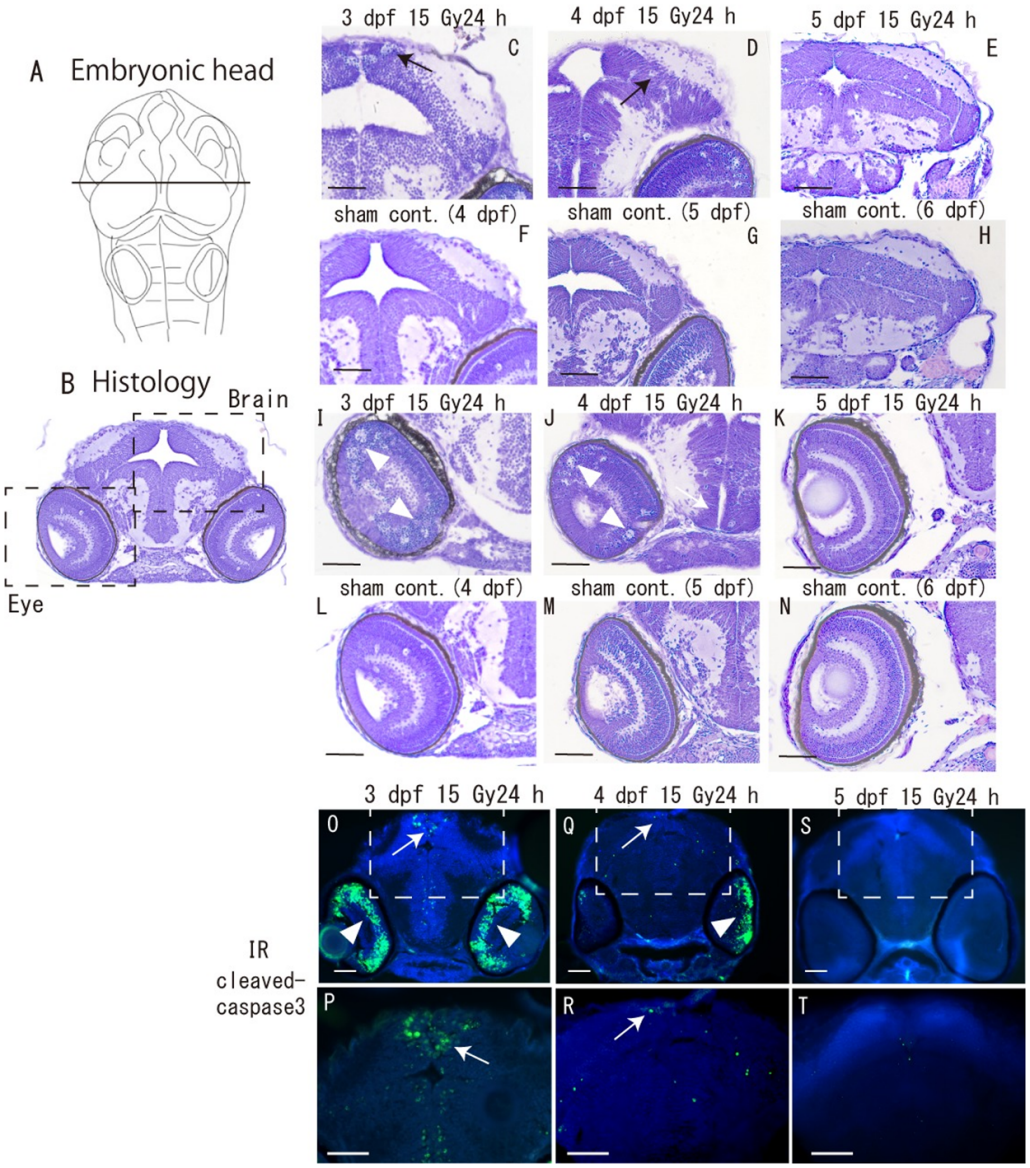
Histological analyses of irradiation-induced apoptotic cells in the brains and eyes during later period of embryogenesis. (A) shows schematic image of embryonic head. Frontal plastic sections (C–N) and cryo-sections (O–T) of the embryonic brain were prepared at the level of the solid line shown in A. Apoptotic cells were examined in the brain and eye (squared area with dotted line in B). Nissl-stained sections of embryos 24 h after irradiation of 3 dpf (C, I) 4 dpf (D, J) and 5 dpf (E, K) and respective controls (F, L), (G, M), and (H, N). Immunohistochemical sections with anti-cleaved-caspase3 of embryos 24 h after irradiation of 3 dpf (O) 4 dpf (Q) and 5 dpf (S). Magnified images in squared area with dotted outlines in O, Q and S are shown in P, R and T, respectively. In irradiated 3 dpf embryos, pyknotic cells were present in the marginal area of the OT (arrow in C) and in the eye (arrowheads in I) and cleaved-caspase 3-positive apoptotic neurons are present in the brain (arrows in O and P) and eyes (arrowheads in O). Fewer apoptotic cells were found in the brain (arrows in D, Q and R) and eyes (arrowheads in J and Q) of irradiated 4 dpf embryos, and no apoptotic cells were found in irradiated 5 dpf embryos (E, K, S and T). No pyknotic cells were present in the brain and eyes of sham controls of 4–6 dpf embryos (F–H and L–N), respectively. Scale bars = 50 μm.

The detailed distributions of apoptotic cells in 3, 4 and 5 dpf embryos 24 h after irradiation were analyzed by Nissl histological staining and immunostaining using an anti-cleaved-caspase 3 antibody. In irradiated 3 dpf embryos, clustered pyknotic cells were present in the marginal area of the OT (arrow in Fig 2C) and in the eye (arrowheads in Fig 2I), which was identical to the distribution of cleaved-caspase 3-positive apoptotic neurons in the brain (arrows in Fig 2O and 2P) and eyes (arrowheads in Fig 2O). The numbers of apoptotic cells in the brain (arrows in Fig 2D, 2Q and 2R) and eyes (arrowheads in Fig 2J and 2Q) were drastically decreased in irradiated 4 dpf embryos, thereafter no apoptotic cells were present in irradiated 5 dpf embryos (Fig 2E, 2K, 2S and 2T). No pyknotic cells were present in the brains and eyes of 4, 5 and 6 dpf sham-control embryos as assessed by Nissl staining (Fig 2F–2H and 2L–2N) and by cleaved caspase 3 immunostaining (data not shown).

**Fig 3:**
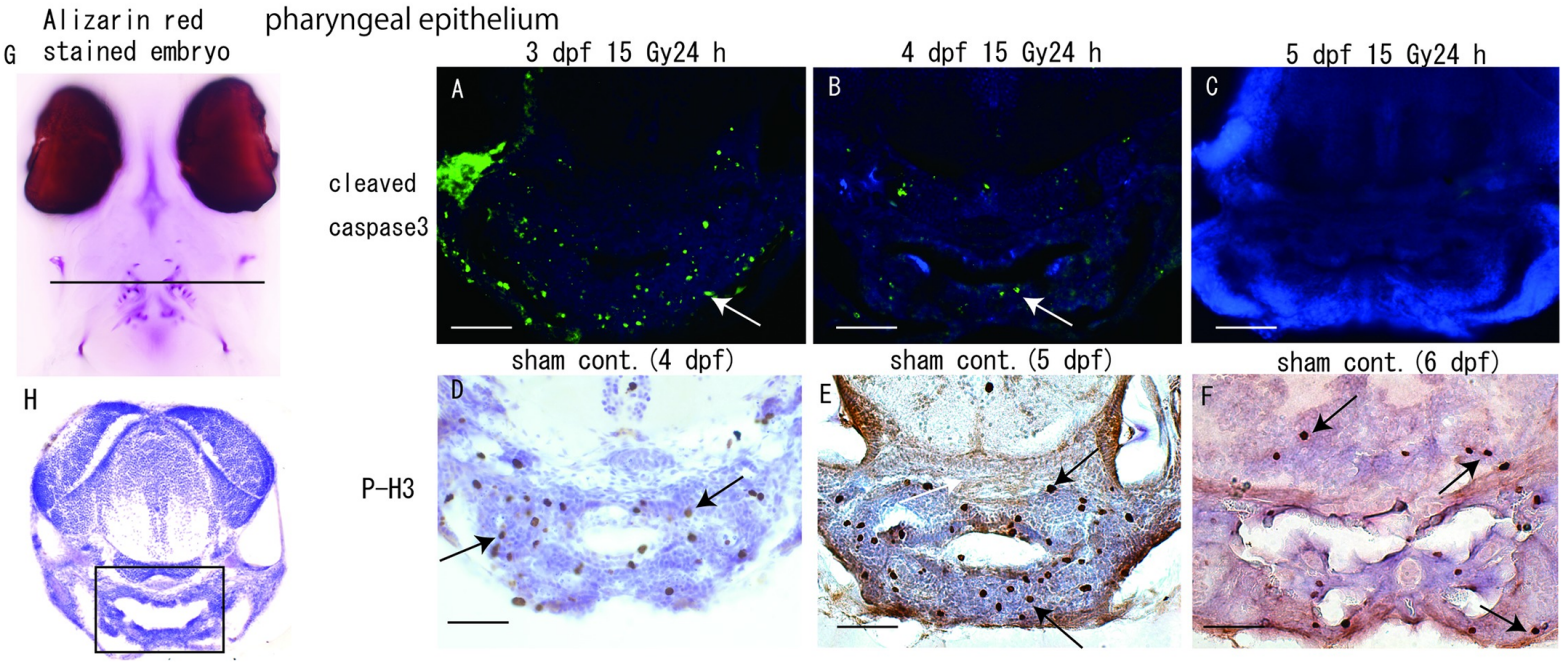
Histological analyses of irradiation-induced apoptotic cells in the pharyngeal epithelium during later period of embryogenesis. (G) shows the image of alizarin red stained embryo at hatching period. Frontal cryo-sections were prepared at the level of the solid line in G (A–F). Immunohistochemical analyses of pharyngeal epithelium tissue (squared area in H) were performed 24 h after irradiation with 15 Gy of gamma rays. Many cleaved-caspase 3-positive cells were present in irradiated 3 dpf embryos (arrow in A), thereafter these signals were relatively fewer in irradiated 4 dpf embryos (arrow in B). No positive signal was evident in irradiated 5 dpf embryos (C). Highly proliferative cells, corresponding to anti-P-H3-positive signals were present in the pharyngeal epithelium of normal development during later embryogenesis, at 4 dpf (D), 5 dpf (E) and 6 dpf (F). Scale bars = 50 μm.

Since the irradiation-induced apoptotic cells in the embryonic brain of medaka were found during a limited period of later embryogenesis, we examined another tissue which is known to be highly proliferative, pharyngeal epithelium. As we expected, immunohistochemical analyses using anti-P-H3 antibody showed a large number of anti-P-H3-positive signals in 4–6 dpf control embryos, respectively (Fig 3D–3F). Immunohistochemical analyses using an anti-cleaved-caspase 3 antibody in 3, 4 and 5 dpf irradiated embryos 24 h after irradiation showed that many cleaved-caspase 3-positive cells were present in irradiated 3 dpf embryos (Fig 3A), thereafter the number of positive signals decreased in irradiated 4 dpf embryos (Fig 3B). No positive signals were found in irradiated 5 dpf embryos (Fig 3C), indicating that, as in the brain tissue, irradiation-induced apoptosis occurred only the restricted period of developmental stages, corresponding to 3 dpf and 4 dpf in the pharyngeal epithelial tissue.

### Cell cycle arrest following irradiation occurred at a limited stage during later period of embryogenesis

**Fig 4:**
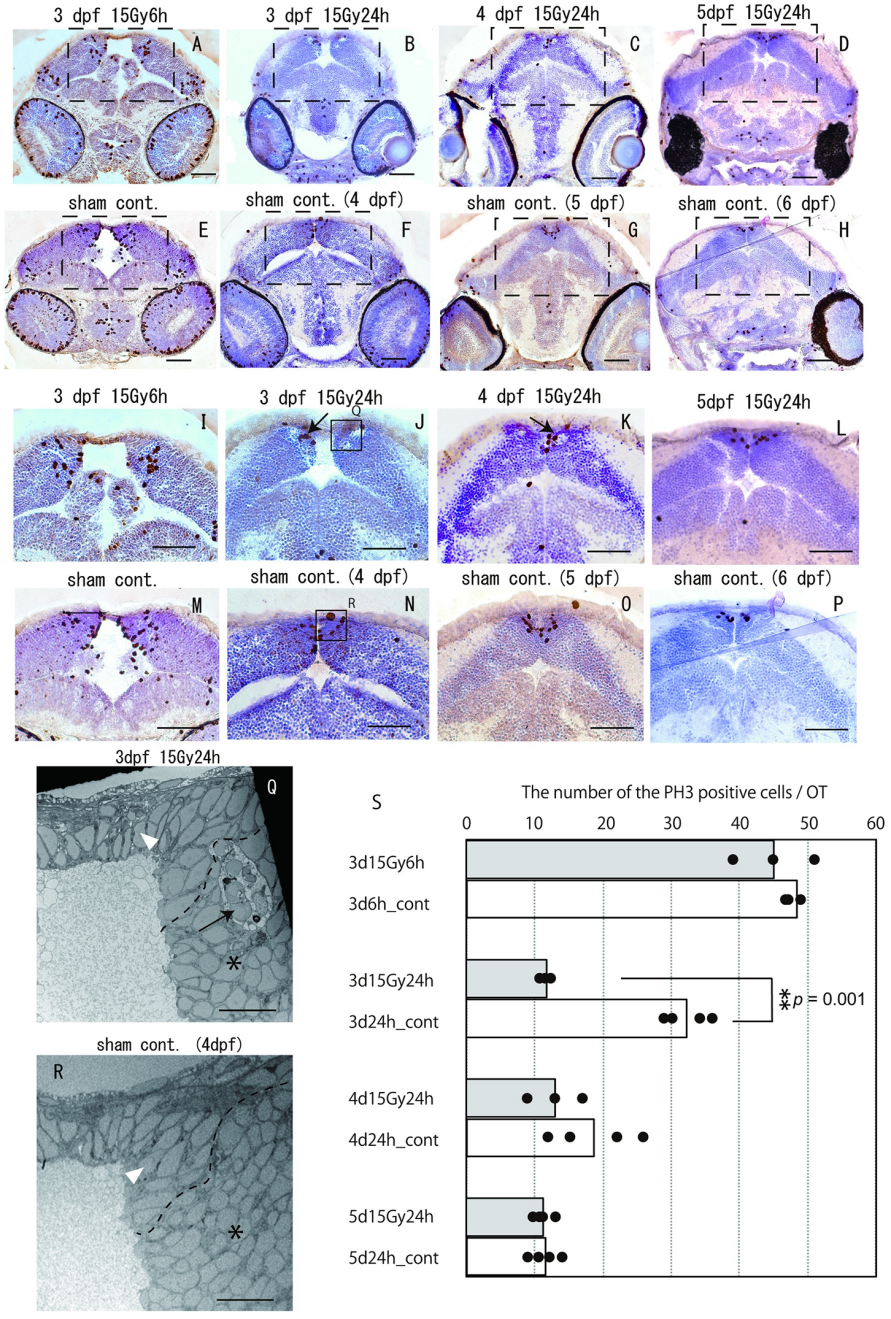
Immuno-stained histology with anti-P-H3 antibody for analyses of proliferating cells in irradiated OT during later period of embryogenesis. Histological sections were stained with anti-P-H3 antibody at 6 h after irradiation in irradiated embryos at 3 dpf (A), and at 24 h after irradiation in irradiated embryos at 3 dpf (B), 4 dpf (C) and 5 dpf (D). Magnified images of the squared area in irradiated embryos (A–D) and their sham controls (E–H) are shown in (I–L) and (M–P), respectively. At 6 h after irradiation, 45.0 ± 6.0 and 48.3 ± 1.15 cells were anti-P-H3 positive in the irradiated 3 dpf embryos and the sham-control embryos, respectively, which was no statistically significant difference (*p* = 0.44) (S). At 24 h after irradiation, the appearance of anti-P-H3-positive cells in the OT was statistically different between irradiated 3 dpf embryos (B, J) and control embryos (F, N) with 11.8 ± 0.50 and 32.3 ± 3.30 cells being positive, respectively (*p* = 0.001) (S). At 24 h after irradiation, 13.0 ± 3.27 and 18.8 ± 5.54 cells were anti-P-H3 positive in irradiated 4 dpf embryos (C, K) and controls (G, O), which was not significantly different (*p* = 0.43) (S). At 24 h after irradiation, 11.3 ± 1.09 and 11.5 ± 1.80 cells were anti-P-H3 positive in irradiated 5 dpf embryos (D, L) and controls (H, P), which was not significantly different (*p* = 0.85) (S). (Q) shows squared area in irradiated 3 dpf embryonic brain (J), and (R) shows squared area of sham-control brain (N). These electron microscopic observations revealed two types of neurons; elongated neurons that localized in the restricted margin of OT (upper area divided with dotted line) (arrowheads in Q, R) and round shaped neurons that occupied most of the area of OT (beneath the area divided with dotted line) (* in Q, R). Apoptotic induction was observed in the area where round shaped neurons were occupied (arrow in Q). Scale bars (A–P) = 50 μm; (Q, R) = 10 μm.

**Fig 5:**
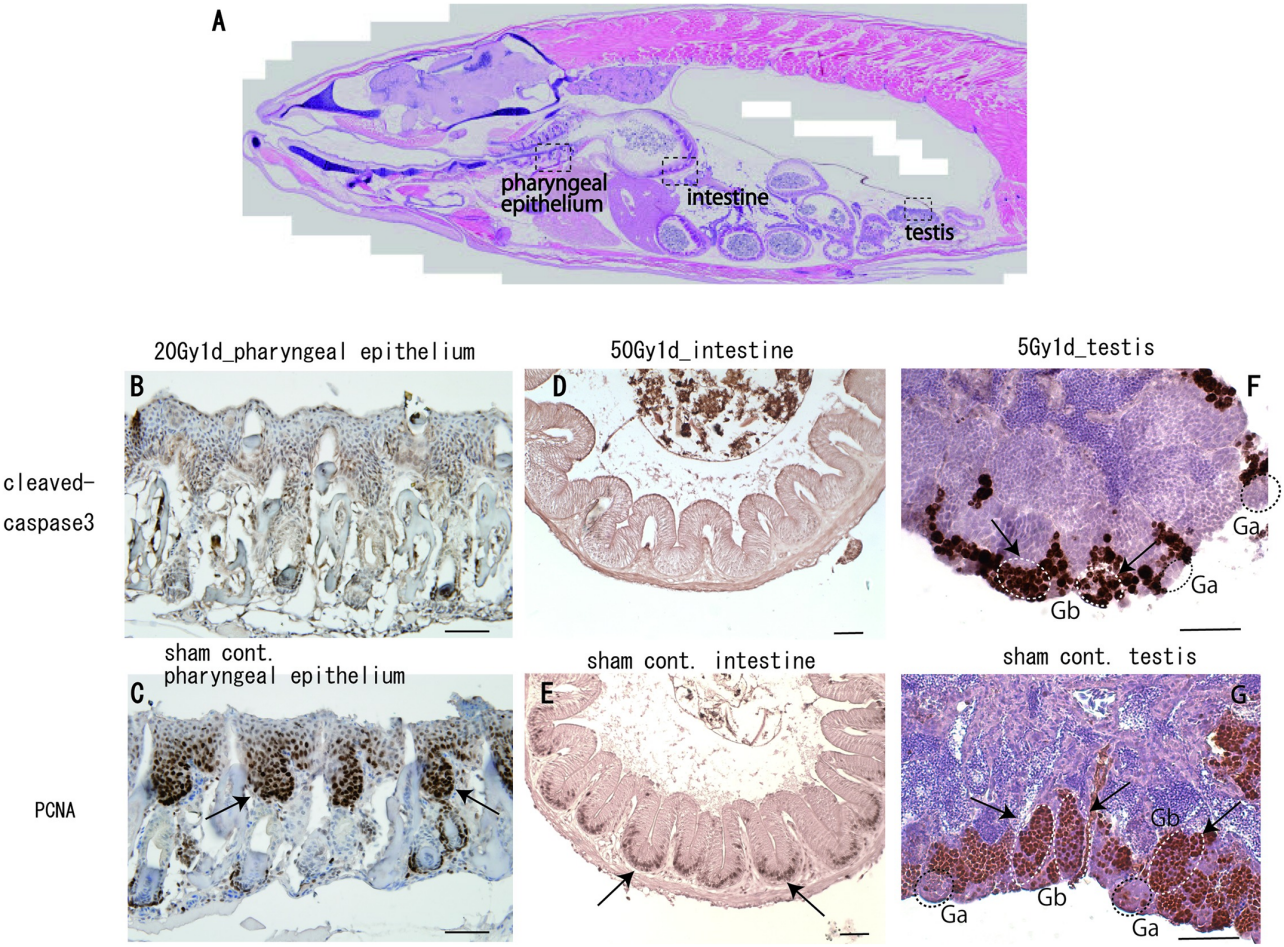
Immuno-histological analyses of irradiation-induced apoptosis in mature proliferating tissues of adult medaka. (A) The sagittal section of whole-body adult medaka as a high-resolution seamless tiling image was cited from a web browser (https://ds.cc.yamaguchi-u.ac.jp/~vs_08_2p/newpage4.html; image from HdrR_M_221.). In mature medaka, actively proliferating tissues including pharyngeal epithelium, small intestine and testis (squared area with dotted outline in A) were examined 24 h after irradiation. PCNA-positive cells were present in a proliferating area of the pharyngeal epithelium (arrows in C), the bottom of intestinal folds (arrows in E) and in cysts of proliferating spermatogonial cells (Gb) in the testicular tissue (arrows in G). In contrast, no cleaved-caspase 3-positive signals were observed 24 h after irradiation in the pharyngeal epithelium (B) and the intestinal tissue (D). In the mature testis, many cleaved-caspase 3-positive cells were present in the Gb cysts (arrows in F) where PCNA-positive proliferating spermatogonial cells are localized (arrows in G), whereas no cleaved caspase 3 -positive cells were found in the Ga cysts, circled area with black dotted outline in F, where no PCNA positive cells were present (circled areas with black dotted outline in G). Scale bars = 50 μm.

It was expected that the cell cycle of irradiated neurons in the OT would be arrested due to irradiation-induced genomic damage. To evaluate the cell cycle frequency of proliferating cells in the OT of irradiated embryos, histological sections were stained with anti-P-H3 antibody 6 h after irradiation in 3 dpf embryos (Fig 4A and 4I), and 24 h after irradiation in 3 dpf (Fig 4B and 4J), 4 dpf (Fig 4C and 4K) and 5 dpf (Fig 4D and 4L) embryos.

There was no apparent difference in proliferating cells in the OT (based on positive number of the anti-P-H3 antibody) at 6 h after irradiation in 3 dpf irradiated embryos, with 45.0 ± 6.0 positive cells (mean ± SD) (n = 3), compared to 48.3 ± 1.15 positive cells in sham control embryos (n = 3). This difference was not statistically significant (*p* = 0.44) (Fig 4S). At 24 h after irradiation, the appearance of anti-P-H3-positive cells in OT was statistically different between irradiated 3 dpf (Fig 4B and 4J) and control embryos (Fig 4F and 4N), with 11.8 ± 0.50 and 32.3 ± 3.30 cells positive for anti-P-H3 in the irradiated (n = 3) and control embryos (n = 4), respectively (*p* = 0.001) (Fig 4S). At 24 h after irradiation, 13.0 ± 3.27 and 18.8 ± 5.54, 11.3 ± 1.09 and 11.5 ± 1.80 cells were anti-P-H3 positive in the irradiated 4 dpf embryos (n = 3) and the sham-control embryos (n = 4) and in the irradiated 5 dpf embryos (n = 4) and the sham-control embryos (n = 4), respectively, with no statistically significant difference between them (*p* = 0.43 and *p* = 0.85, respectively) (Fig 4S). Furthermore, some anti-P-H3-positive cells were localized close to rosette-shaped aggregates of apoptotic cells in the irradiated OT (arrows in Fig 4J and 4K), suggesting that cell proliferation was not suppressed around an area of clustered apoptotic cells. Electron microscopic observation of irradiated 3 dpf embryonic brains at 24 h after irradiation showed that there were two different morphological types of neurons; elongated neurons in the limited area of marginal OT near the torus longitudinal (arrowheads in Fig 4Q and 4R) and spherical shaped neurons occupying a large part of neurons in OT (asterisks in Fig 4Q and 4R). Apoptotic neurons were induced in the area where round shaped neurons were occupied (arrow in Fig 4Q), while there was no evidence of apoptotic induction in the area where elongated neurons were occupied. Of the embryos irradiated at 3 dpf, 100% (26/26) were dead within 3 days after hatching with severe malformations, such as smaller brain and eyes. In contrast, of the embryos irradiated at 5 dpf, 77% (27/35) survived normally with no apparent malformations, similar to the hatchability in control embryos 81% (39/48). Of the embryos irradiated at 4 dpf, 86% (39/45) were dead within 1 week after hatching.

### Irradiation-induced apoptosis did not occur in the pharyngeal epithelium and intestinal tissue but occurred in testicular tissue of adult medaka

Irradiation-induced apoptotic cells were only evident during a limited period of embryogenesis even in actively proliferative tissues of the brain and pharyngeal epithelium. The developing embryonic brain is substantially more susceptible to radiation than mature brain in adult as described in introduction section. In practice, in the adult mature brain of medaka, no cleaved-caspase 3 positive cells were found 24 h after irradiation (data not shown). To confirm whether apoptotic induction was occurred following irradiation in other tissues which are known to be highly proliferative and sensitive to radiation, we assessed apoptosis by immune-histological analyses in tissues including pharyngeal epithelium, intestine and testis. In control tissues, a high number of PCNA-positive cells were present in the tooth replacement region of the pharyngeal epithelium (arrows in Fig 5C), in the bottom region of intestinal folds (arrows in Fig 5E and S1 Fig) and in differentiating spermatogonial cells in the Gb cysts of the testicular tissue (arrows in Fig 5G). These results demonstrated that they are highly proliferating tissues. However, cleaved-caspase 3-positive signals were not evident 24 h after irradiation in pharyngeal teeth (Fig 5B) and small intestine (Fig 5D). In contrast, in testicular tissue, a large number of cleaved-caspase 3-positive cells were present in Gb cysts (arrows in Fig 5F), where PCNA-positive proliferating spermatogonial cells are present (arrows in Fig 5G), which was consistent with our previous results [29]. Whereas no cleaved caspase 3 -positive cells were present in the Ga cysts (circled areas with black dotted outline in Fig 5F), where no PCNA positive cells were present (circled areas with black dotted outline in Fig 5G).

## Discussion

**Fig 6:**
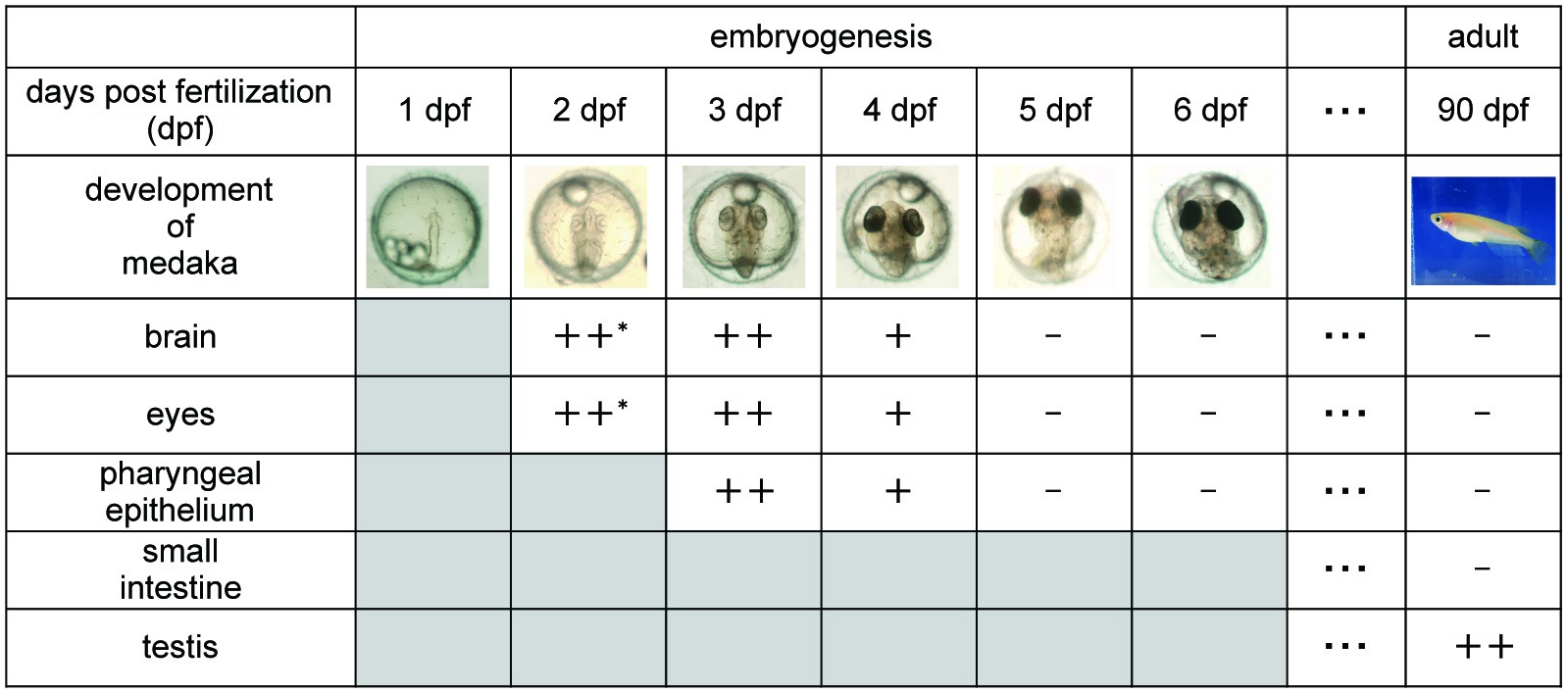
The occurrence of apoptotic cells in various medaka tissues throughout the lifespan. The occurrence of apoptotic cells in the brain, eyes, pharyngeal epithelium, small intestine and testis is represented as follows: ++ many apoptotic cells, + few apoptotic cells and—no apoptotic cells could be identified. The results with asterisks have been reported previously by Yasuda *et al*. 2006. Grey coloured columns indicate that the tissue is not sufficiently developed to identify apoptotic cells.

Our previous study showed that medaka embryos irradiated at 3 dpf with 10 Gy of gamma rays exhibited many apoptotic cells in the OT. The irradiated embryos hatched and survived; however, they had smaller brains, and the histological results concerning retinal arrangements were abnormal. To identify the severity of biological effects following irradiation during later periods of embryogenesis, embryos at various developmental stages were irradiated with a high dose of gamma rays (15 Gy) and examined for apoptotic induction at 24 h after irradiation. Consequently, we found that irradiation-induced apoptosis occurred during a limited period of embryogenesis, corresponding to 3 dpf and 4 dpf, with the exception of testicular tissue, in which apoptosis occurred throughout the life stages (Fig 6).

In pharyngeal epithelium of adult medaka, the existence of odontogenic stem cell niches that can continuously generate and replace teeth throughout the lifespan [32–34]. Unexpectedly, irradiation-induced apoptotic cells were not found in actively proliferating tissues of matured pharyngeal epithelium and intestine of adult medaka. Mammalian intestinal cells are highly proliferative tissue and cells of intestine are susceptible to irradiation damage [35]. In mammals, the base of each villus is surrounded by multiple epithelial invaginations, termed crypts, where vigorously proliferating stem cells involved in epithelium self-renewal reside. Mouse intestinal stem cells consist of two populations: slow-cycling quiescent stem cells and actively cycling stem cells, which are responsible for regeneration following irradiation-induced injury [36, 37]. Transplantation of mesenchymal stem cells has been shown to reduce the number of apoptotic cells and improve the survival rate of irradiated mice [38], suggesting that a different mechanism could be involved, based on the structural differences of stem cells, in intestinal regeneration following irradiation-induced injury. This previous report suggests that the structural differences of resident intestinal stem cells in medaka (S1 Fig) and mice could be associated with the differences in the occurrence of apoptosis. In addition, the survival period of medaka following irradiation increased as water temperatures decreased [39], suggesting that slower cell cycles induced by lower temperature could attenuate intestinal injury-induced mortality following irradiation. This result strongly suggested that irradiation-induced apoptosis in the intestine of poikilothermic medaka could be attenuated due to the slower cell cycle than homeothermic mammals.

In embryonic brain of medaka, irradiation induced apoptosis occurred during a limited period of embryogenesis, corresponding to 3 dpf and 4 dpf. Electron microscopic observations demonstrated two different morphological types in the marginal area of the OT; the majority of cells had spherical nuclei, whereas the remaining cells had elongated fusiform nuclei and were in a specific marginal area of the OT (Fig 4Q and 4R). Juvenile and adult medaka have two discrete cell populations: slow-cycling stem cells, which have elongated fusiform nuclei and are located in only in the margin of the OT, and actively proliferating cells, which are round-shaped cells located within the centre of the OT [40–42]. Based on these results, the elongated fusiform cells in the marginal area of the embryonic OT in this study might be slow-cycling neuronal stem cells, whereas the cells with spherical nuclei adjacent to the elongated fusiform cells might be anti-P-H3-positive, actively proliferating progenitor cells. Our present electron microscopic observations demonstrated that irradiation-induced apoptosis did not occur in the cells with elongated fusiform nuclei but occurred in the spherical nuclei cells, suggesting that neuronal stem cells are resistant to irradiation, while proliferating progenitor cells are sensitive to irradiation. Because the proportion of anti-P-H3-positive progenitor cells in the restricted margin of the OT relative to all neuronal cells in the OT sharply decreased as embryogenesis proceeded (Fig 4M–4P), the frequency of apoptosis following irradiation also drastically decreased, as shown in Figs 1 and 2.

In testicular tissue of medaka, two different populations of stem cells: slow-cycling, quiescent stem cells, termed Ga, and actively differentiating stem cells, termed Gb, that are classified by cyst architecture. In the mature testis of medaka, as in brain tissue, apoptosis was rarely observed among the slow-cycling stem cells, in the Ga cysts. Many cleaved-caspase 3-positive apoptotic cells were found in adult testicular tissue in the cysts of actively proliferating spermatogonial cells (Gb) (Fig 5F and 5G).

Stem cells are defined as undifferentiated cells that can self-renew through mitotic cell division to replenish stem cell pool and differentiate into other cell types essential for tissue function [43–45]. Both embryonic and adult stem cells rely on a very robust DNA damage response, which is beneficial as it preserves optimal stem cell function in healthy tissues [46, 47]. Embryonic stem cells (ESCs) repair various lesions much more efficiently than differentiated cells, in addition, ESCs preferentially employ homologous recombination which is an error-free DSB repair system over non-homologous end joining, hence repairing DSBs with increased fidelity [48, 49]. In accordance with these previous reports, our results in medaka demonstrated that stem cells of embryonic brain and of adult testicular tissue in Ga cyst were resistant to irradiation, possibly owing to recovery from radiation-induced injury which is critically dependent on the repopulation of resident stem cells.

## Supporting information

S1 Fig The internal structure of non-irradiated medaka small intestine. (A) External appearance and internal structure of small intestine (a, c). Scanning electron microscope images of internal small intestine (b, d). (B) Mature medaka intestine; PCNA-positive cells on serial histological sections (a) were segmented manually as colored spots, (b) segmented images stacked as X–Y–Z images and (c) reconstructed 3D images of PCNA-positive cells (d). (C) Non-irradiated medaka intestine; (a) PCNA-positive cells are represented as red spots in 3D images (b–d). 3D images of sideways section and (e, f), 3D images of lengthwise section (c, d, f) images in(c), (d), and (f) are from the direction of the white arrow in (e). Scale bars (b, d) = 50 μm; (c) = 250 μm.
(DOCX)
