# Supplementary material for: Radical change of apoptotic strategy following irradiation during later period of embryogenesis in medaka (Oryzias latipes)
Source: PLoS One. 2018 Aug 3;13(8):e0201790. doi: 10.1371/journal.pone.0201790 (PMC6075778; doi:10.1371/journal.pone.0201790)
Supplement: S1 Fig — (A) External appearance and internal structure of small intestine (a, c). Scanning electron microscope images of internal small intestine (b, d). (B) Mature medaka intestine; PCNA-positive cells on serial histological sections (a) were segmented manually as colored spots, (b) segmented images stacked as X–Y–Z images and (c) reconstructed 3D images of PCNA-positive cells (d). (C) Non-irradiated medaka intestine; (a) PCNA-positive cells are represented as red spots in 3D images (b–d). 3D images of sideways section and (e, f), 3D images of lengthwise section (c, d, f) images in(c), (d), and (f) are from the direction of the white arrow in (e). Scale bars (b, d) = 50 μm; (c) = 250 μm. (DOCX) [file pone.0201790.s001.docx]

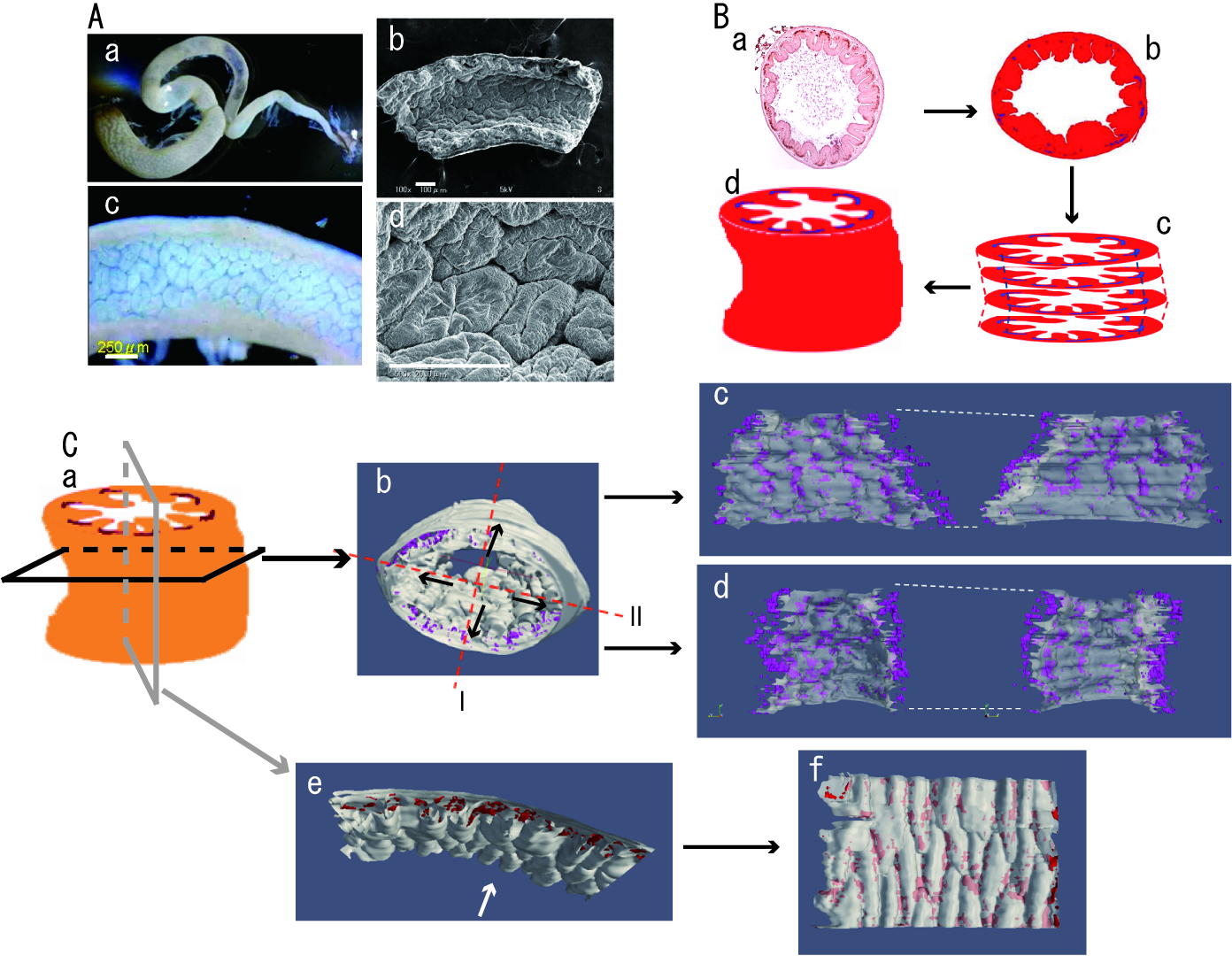


**S1 Fig. The internal structure of non-irradiated medaka small intestine**

(A) External appearance and internal structure of small intestine (a, c). Scanning electron microscope images of internal small intestine (b, d). (B) Mature medaka intestine; PCNA-positive cells on serial histological sections (a) were segmented manually as colored spots, (b) segmented images stacked as X–Y–Z images and (c) reconstructed 3D images of PCNA-positive cells (d). (C) Non-irradiated medaka intestine; (a) PCNA-positive cells are represented as red spots in 3D images (b–d). 3D images of sideways section and (e, f), 3D images of lengthwise section (c, d, f) images in(c), (d), and (f) are from the direction of the white arrow in (e). Scale bars (b, d) = 50 μm; (c) = 250 μm.
